# Follow-up of children and adolescents with short stature: the importance of the growth rate

**DOI:** 10.1590/S1516-31802005000300008

**Published:** 2005-05-02

**Authors:** Maria Wany Louzada Strufaldi, Edina Mariko Koga da Silva, Rosana Fiorini Puccini

**Keywords:** Growth disorder, Body height, Child, Adolescent, Growth, Transtornos do crescimento, Estatura, Criança, Adolescente, Crescimento

## Abstract

**CONTEXT AND OBJECTIVE:**

Short stature is defined as a height of more than two standard deviations below the average for a given age and sex in a reference population. The objective was to describe follow-up conducted among short-stature children and adolescents.

**DESIGN AND SETTING:**

Descriptive study, at the Growth outpatient clinic, Department of Pediatrics, Universidade Federal de São Paulo.

**METHODS:**

The study included 152 patients aged 2 to 15 years who had height for age of less than P5, on the National Center for Health Statistics curve. The children underwent nutritional evaluation, and several variables relating to height and growth rate were calculated to establish etiological diagnosis. Bone age was evaluated by X-ray.

**RESULTS:**

The majority (63.2%) were male. In 77.8%, the stature observed was within the family pattern. Among the 99 patients followed up for more than 6 months, 17.2% presented inadequate growth rates. The preponderant etiological diagnosis for short stature was familial/constitutional in 58.6% of the cases; 27 patients (34.2%) with adequate growth rate presented bone age alterations. Even with inadequate growth rates, 75% of such patients had a normal result from growth hormone stimulation testing. Close to 90% of patients with a diagnosis of short stature of familial/constitutional origin and intrauterine growth retardation presented adequate growth rate. The genetic etiology was significantly characteristic of patients with inadequate growth rate.

**CONCLUSION:**

Growth rate assessment must form part of the investigation and follow-up of short-stature cases. However, its utilization and validity should form part of an overall view of each patient.

## RESUMO

**CONTEXTO E OBJETIVO:**

Baixa estatura é definida como uma altura abaixo de dois desvios-padrão da média para uma determinada idade e sexo numa população de referência. O objetivo do estudo foi descrever o acompanhamento de crianças e adolescentes com baixa estatura.

**TIPO DE ESTUDO E LOCAL:**

Estudo descritivo, no Ambulatório Crescer, Departamento de Pediatria, Universidade Federal de São Paulo.

**MÉTODOS:**

Foram incluídos 152 pacientes com idade de 2 a 15 anos e estatura para idade menor que o percentil 5, segundo a curva do National Center for Health Statistics (NCHS). As crianças passaram por avaliação nutricional e diversas variáveis relacionadas com altura e velocidade de crescimento foram calculadas para estabelecimento de diagnóstico etiológico. A idade óssea foi avaliada por raio-x.

**RESULTADOS:**

A maioria (63,2%) era do sexo masculino. Em 77,8%, a estatura observada encontrava-se dentro do canal familiar. Dentre os 99 pacientes com período de seguimento superior a seis meses, 17,2% apresentaram velocidade de crescimento inadequada. O diagnóstico etiológico preponderante da baixa estatura foi familiar/constitucional em 58,6% dos casos. 27 pacientes (34,2%) com velocidade adequada, apresentaram raios-X de osso alterados. Mesmo com velocidade inadequada de crescimento, em 75% dos pacientes com teste de estímulo de GH, o resultado foi normal. Próximo de 90% dos pacientes com diagnóstico de baixa estatura de origem familiar/constitucional e restrição do crescimento intra-uterino, demonstraram velocidade de crescimento adequada. A etiologia genética caracterizou significantemente, pacientes com velocidade inadequada de crescimento.

**CONCLUSÃO:**

A velocidade de crescimento deve fazer parte da investigação e do acompanhamento da baixa estatura, porém, sua utilização e validade deveriam fazer parte da análise de uma visão global de cada paciente.

## INTRODUCTION

In following up growth during childhood, the healthcare professional (generally a pediatrician) is almost always faced with the normal variations within this process, and with diseases that compromise the child's weight-height evolution. Despite the favorable progression in most cases, children of short stature can benefit from the advances in diagnosis and therapy in some specific situations. In this light, it is important for the pediatrician to keep up-to-date and familiarized with the potential benefits of early diagnosis and treatment. At the same time, in view of the complexity of the diagnosis and the numerous etiological possibilities, it is impracticable to investigate all the possibilities for all children.

Short stature is defined as a condition in which an individual has a height that is more than two standard deviations below the average height for a given age and sex in a reference population. Whether a child's stature is "abnormal" purely from a statistical point of view, or whether it is indicative of inadequate growth, is a question that needs to be defined by additional criteria.^1^

According to a World Health Organization bulletin published in 2000, the prevalence of children with height deficit, defined as height for age that is more than two standard deviations below the reference, has diminished over the last 20 years. In developing countries, around 32.5% of children present such deficit (stunting). More specifically in South America, there was a fall in this prevalence from 25.1% to 9.3%, over the period from 1980 to 2000.^2^

The present study arose from the need to analyze the attendance provided for children and adolescents at an interdisciplinary outpatient clinic dealing with short stature. Its aim was both to contribute towards optimizing diagnoses and to reinforce the actuation of general pediatrics in following up such children. Thus, the growth outpatient clinic was created in 1997, with the participation of professionals from the disciplines of community pediatrics, endocrinology, genetics and nutrition and metabolism.

## PATIENTS AND METHODS

All the children and adolescents aged two years or over, who were enrolled during the period from March 1997 to April 2001, were included for follow-up at this interdisciplinary outpatient clinic if they presented a height of less than P5 on the National Center for Health Statistics (NCHS) curve, which was chosen as the reference.

The height measurement was always performed with the child or adolescent standing erect, without shoes, undressed and with the heels together, arms resting at the side of the body and the head positioned parallel to the floor. The patients were weighed on a digital Filizola^®^ balance.

For the nutritional evaluation of patients aged less than 10 years, the weight for height was calculated and those with results of less than 90% were considered to be malnourished. For patients aged 10 years or over, the body mass index was calculated and those whose body mass index was less than P5 on the NCHS curve were considered to be malnourished.^3,4^

The target height was calculated from the average stature of the parents, plus 6.5 cm for boys and minus 6.5 cm for girls, with upward and downward variation of two standard deviations.^5^

The z-score for the stature of each child was calculated by subtracting the child's height from the average expected for the age and sex, in accordance with the reference curve, and then dividing the result of this subtraction by the standard deviation corresponding to the average expected. The z-score of the parents' average height was also calculated and compared with the initial z-score for the parents' height. Z-scores for patients that were below the lower limit of the parents' z-score were considered to be abnormal or outside of the family's pattern.

The following parameters were calculated via the SISCRES (System for Analysis of Data and Growth) program^6^: initial z-score for stature, final z-score for stature (relating to the last height measurement made at the outpatient clinic), body mass index, growth rate (cm/year), target height, z-score for the parents' average height and their limits.

The following criteria were utilized in defining the etiological diagnosis:

- Familial: short stature within the average predicted from the parents' stature; bone age in accordance with chronological age (within —2 standard deviations); growth rate adequate for the age; absence of delay in puberty.
- Constitutional: short stature in patients with stature or growth pattern below the prediction from the parents' stature; delay in puberty and/or bone age; inadequate growth rate before the peak of the pubertal stretching; positive family history of delayed puberty.
- Intrauterine growth retardation: short stature in patients whose birth weight was inadequate for the gestational age. Since for most patients the gestational age cannot be precisely ascertained, it was considered that patients born with weights of less than 2,500 g who were reported to have been delivered at full term should be included within this criterion. The choice of this cutoff point is justifiable because, according to Alexander's curve, all newborns with more than 37 weeks of gestation and weight of less than 2,500 *g* are situated below the P10 of this curve.^7^
- Hormonal: short stature in patients with deficient peak response to two growth hormone stimulation tests, or abnormal thyroid function. Patients were considered to have responded to the growth hormone stimulation test, in relation to the different stimuli (exercise, clonidine and insulin hypoglycemia), if there was a peak of greater than 10 ng/dl or Δ > 7 ng/dl (difference between the maximum and base values).^8-10^ A diagnosis of hypothyroidism was made for patients with lowT4 and high thyroidstimulating hormone (TSH) levels, in accordance with the reference utilized.
- Genetic: short stature in patients with the presence of phenotypic variations, wrongly proportioned bodies or characteristic chromosomal alterations.
- Secondary to chronic diseases: short stature in patients with chronic illnesses that justify the height deficit. It is emphasized that, in treating asthmatic patients, such patients were only included within this criterion if they had asthma associated with the chronic use of corticoid therapy (oral or inhalatory).

The bone age was assessed according to radiography of the left hand and wrist, utilizing the Greulich and Pyle patterns. It was characterized as abnormal if the patient presented a bone age that was more than two standard deviations lower than expected for the chronological age and sex. Other complementary examinations were only requested according to the individual case.

After a minimum period of six months following the first attendance, it was possible to calculate the patient's growth rate, based on the subtraction of the observed height from the height at the first consultation. Growth rates of less than 4 cm per year for patients who had not yet reached puberty were considered to be inadequate, and rates below P3 on the Tanner growth rate curve were considered inadequate for patients whose puberty had already started.^11^ Another way of evaluating the growth rate was via the difference in z-score (final z-score minus initial z-score), with a growth rate of ≤ —0.75 considered inadequate.^12^

### Statistical analysis

To compare the category variables, the chi-squared test was utilized, calculated via the Epitable program from EpiInfo 6.01.^13^ For calculating and comparing the averages, the variance analysis method was utilized (by means of the same program). For all the statistical tests, a significance level of 5% was adopted (μ = 0.05). When the calculated p value (minimum significance level) allowed the nullity hypothesis to be rejected, an asterisk was utilized to identify such a finding (p < 0.05).

## RESULTS

From the 152 children and adolescents who began follow-up, 23 patients (15.1%) were only present at one consultation and 30 (19.7%) were followed up for less than 6 months. Among the remaining 99 patients who were followed up for 6 months or more, 34 (34.3%) were followed up for at least two years.

Upon enrolling in the outpatient clinic, the patients' ages ranged from 2 to 15 years, with an average of 8.6 years, and the majority were male (63.2%). Among both male and female patients, there was no predominance of any age group for the age of enrolment in the outpatient clinic.

It was possible to measure the heights of 94.7% of the mothers of the children and adolescents in the study. The mothers' average height was 153.6 cm, with standard deviation of 6.6 cm. The father's height was measured in 90.7% of the cases, with an average of 165.9 cm and standard deviation of 7.4 cm. For 52.8% of the mothers and 34.7% of the fathers, their heights were less than P5, according to the NCHS curve.

Since the follow-up was fundamental for defining the etiology of short stature, the study only took into consideration the children and adolescents who continued with the follow-up for a minimum period of six months, thus totaling 99 patients.

The etiology of the short stature was considered to be the predominant cause observed after this follow-up period, with short stature of familial and/or constitutional cause grouped as a single diagnostic item. Each child or adolescent was characterized with a main diagnosis for the short stature, with the exception of one child who had Stickler syndrome with growth hormone deficiency, for whom it was considered pertinent to maintain two diagnoses. Thus, it is stressed that although the subsequent analyses were done on 99 patients, there were 100 main etiological diagnoses (Table 1).

**Table 1:** Etiological diagnosis for the short stature of 99 patients followed up at a growth outpatient clinic for six months or more in São Paulo, 1997-2001

Etiology	No. of diagnoses	%
Familial/constitutional	58	58.0
Hormonal	02	2.0
Intrauterine growth retardation	14	14.0
Genetic	09	9.0
Secondary diseases	04	4.0
Ongoing investigation	13	13.0
**Total** *	**100** *	**100.0**

* *One patient had two diagnoses (hormonal + genetic).*

A majority of the patients (50.7%) with the diagnoses analyzed presented an initial z-score for stature of between -2 and —2.99, which was statistically significant for the etiology of familial/constitutional cause. One patient with a height of less than -4 z-scores presented low stature of familial/constitutional origin.

Among the 88 patients who continued with follow-up for more than six months and whose parents had their heights measured, 13 presented an initial z-score that was below the lower limit for prediction of stature. Of these, five patients had short stature of constitutional cause, three presented intrauterine growth retardation, three presented short stature of genetic origin, one presented a hormonal cause and two were still undergoing diagnostic investigation (As mentioned above, one child had two different diagnosis).

Predominance of males with familial/constitutional and genetic etiologies was observed, and it is emphasized that all the patients with hormonal cause and origins in secondary diseases were also male.

It was seen that the majority of the patients with a hormonal and genetic diagnosis presented ages between five and 10 years. It was also seen that almost half (46.6%) of the patients with a familial/constitutional cause were adolescents.

With regard to the birth weight of the children and adolescents in the study and its relationship with the etiology for the short stature, a significant predominance of weight greater than or equal to 2,500 g was observed among those with a familial/constitutional cause and, as predicted, all the patients with intrauterine growth retardation significantly presented low weight or inadequate birth weight (Table 2).

**Table 2:** Etiological diagnosis for the short stature of patients followed up at a growth outpatient clinic for six months or more, according to birth weight, in São Paulo, 1997-2001

Etiology	< 2,500 g		2,500-2,999 g		≥ 3,000 g		Total	
	n	%	n	%	n	%	n	%
Familial/constitutional*	06	11.8	16	31.4	29	56.9	51	100.0
Hormonal	–	–	–	–	02	100.0	02	100.0
lUGR†	12	85.7	02	14.3	–	–	14	100.0
Genetic	04	44.4	02	22.2	03	33.3	09	100.0
Secondary diseases	01	33.3	01	33.3	01	33.3	03	100.0
Ongoing investigation	06	60.0	02	20.0	02	20.0	10	100.0
**Total** ‡							**88**	**100.0**

* *p<0.01; IUGR: intrauterine growth retardation;*

† *p < 0.01.*

‡ *11 patients (11.1%) without birth weight information.*

Among the 99 patients followed up for more than six months, nutritional inadequacy was observed in 34 of them (weight/height < 90% or body mass index < P5). Of these, the majority were male (61.8%) and aged less than 10 years (73.5%), with adequate growth rates (85.3%). In addition to this, 43.3% presented birth weights of less than 2,500 g.

The growth rate in cm/year for patients attending the outpatient clinic was considered to be inadequate in 17.2% of them. Of these, the majority (82.4%) were males, corresponding to 21% of the children who had not reached and 10.8% of the adolescents undergoing puberty. Only two patients (one child and one adolescent) presented an abnormal z-score difference (< -0.75), which made it impossible to proceed with an analysis following this criterion.

Among the 13 patients who had not undergone puberty and had growth rates of less than 4 cm/year, seven did not complete one year of follow-up. Among the adolescents undergoing puberty with an abnormally low growth rate, the majority continued with follow-up for a period of more than three years.

In analyzing the growth rate in relation to the patients' birth weights, no trend or predominance of adequate or inadequate rate was observed for any of the weight criteria adopted.

The X-ray result for evaluating the bone age was considered to be normal for 65.8% of the patients with an adequate growth rate. However, it was observed that 27 patients (34.2%) with an adequate growth rate presented an X-ray for bone age assessment that was outside the range of normality (Table 3).

**Table 3:** Growth rate (cm/year) among patients followed up at a growth outpatient clinic for more than six months, and X-ray results for assessing bone age, in São Paulo, 1997-2001

Growth rate	Normal bone age		Abnormal bone age		Total	
	n	%	n	%	n	%
Adequate	52	65.8	27	34.2	79	100.0
Inadequate*	05	38.5	08	61.5	13	100.0
**Total** †	**57**		**35**		**92**	**100.0**

* *Growth rates of less than 4 cm/year for non-pubertal patients and less than P3 on the Tanner curve (in accordance with sex and age) for pubertal patients were considered inadequate.^11^*

† *7 patients (7.7%) without X-ray for assessing bone age.*

Among the children and adolescents followed up for at least six months who underwent a first growth hormone stimulation test, around 80% of those with a responsive result (> 10 ng/dl or Δ > 7 ng/dl) presented an adequate growth rate. On the other hand, even with an inadequate growth rate, a normal stimulus test result was observed with 75% of the patients. When evaluating the patients who underwent a second stimulus test with the growth rates they presented, a situation similar to that of the first test was found (Table 4).

**Table 4:** Growth rate (cm/year) among patients followed up at a growth outpatient clinic for more than six months, and results from two growth hormone stimulus tests, in São Paulo, 1997-2001

		Test 1					Test 2				
Growth rate	Normal		abnormal		total	Normal†		abnormal		total
	n	%	n	%		n	%	n	%	
Adequate	21	80.8	05	19.2	26 (100.0)	04	80.0	01	20.0	05 (100.0)
Inadequate*	06	75.0	02	25.0	08 (100.0)	03	75.0	01	25.0	04 (100.0)
**Total** ‡	**27**		**07**			**07**		**02**		

* *Growth rates of less than 4 cm/year for non-pubertal patients and less than P3 on the Tanner curve (in accordance with sex and age) for pubertal patients were considered inadequate.^11^*

† *The growth hormone test was considered to be normal with a peak > 10 ng/dl or Δ > 7 ng/dl.*

‡ *Number of patients followed up for six months or more who did the growth hormone test.*

Close to 90% of the patients with a diagnosis of short stature of familial/constitutional origin and intrauterine growth retardation presented an adequate growth rate. The patients with short stature of familial/constitutional etiology displayed a significant statistic difference in relation to the others etiologies. The genetic etiology was significantly characteristic of patients with an inadequate growth rate. There was also one patient with short stature of hormonal cause who presented adequate growth rate (Table 5).

**Table 5:** Etiological diagnosis for the short stature of 99 patients followed up at a growth outpatient clinic for more than six months, according to the growth rate (cm/year), in São Paulo, 1997-2001 (one patient had two diagnosis)

Etiology	Adequate growth rate		Inadequate growth rate*		Total	
	n	%	n	%	n	%
Familial/constitutional†	53	91.4	05	8.6	58	100.0
Hormonal	01	50.0	01	50.0	02	100.0
IUGR	13	92.9	01	7.1	14	100.0
Genetic‡	04	44.4	05	55.6	09	100.0
Secondary diseases	02	50.0	02	50.0	04	100.0
Ongoing investigation	09	69.2	04	30.8	13	100.0
**Total**					**100**	**100.0**

† *p < 0.01; IUGR: intrauterine growth retardation;*

‡ *p < 0.01.*

* *Growth rates of less than 4 cm/year for non-pubertal patients and less than P3 on the Tanner curve (in accordance with sex and age) for pubertal patients were considered inadequate.^11^*

## DISCUSSION

The majority of cases of short stature relate to causes considered to be variations from normality. This was observed from the literature and in the present study, thus highlighting the importance of follow-up for such patients and making invasive investigation unnecessary. On the other hand, the extensive list of etiologies that can lead to short stature as the only clinical manifestation, including causes that can be treated and require various resources for their elucidation, poses a variety of questions for the pediatrician. These include: What are the minimum tests for investigating short stature? At what point in time should they be requested? Should all such patients undergo the same protocol?

We consider these points relevant, because the definitions of short stature found in the literature can be extremely variable, such as heights of less than P2.5, P3 or P5, or a height/age index < -1 standard deviation or < -2 standard deviations for the age and sex, according to the reference curve utilized. There may even be the use of different reference curves, for example the Tanner curve or the NCHS curve.^14-17^ In addition to this, studies of the causes of short stature have been concentrated mainly on endocrinology services or population surveys. In both the latter cases, it is clear that the objectives are different and, in this light, the evaluation of the prevalence of each etiology must be approached with caution.

The etiologies for short stature among the children and adolescents followed up at the growth outpatient clinic for a minimum period of six months did not present major surprises when compared with studies with similar purposes. The majority of patients (around 60%) had a familial/constitutional cause, thus reinforcing how important it is for the general pediatrician to initiate the investigation and perform a large part of the follow-up for children of short stature. We considered that the percentage of cases (14%) in which intrauterine growth retardation was indicated as the main cause of short stature was high. This gives evidence of the importance of the birth weight factor in the evaluation and follow-up of such patients.

In the present study, the criterion for defining intrauterine growth retardation was fundamentally based on the birth weight and gestational age. The concepts of low birth weight, small for gestational age and intrauterine growth retardation have thus come to overlap each other, with subtle differences in the evaluation of patients. Furthermore, it has to be remembered that the genetic causes of short stature may also have determined that the birth weight will be low, which contributes towards the difficulty of specifically identifying the interference of this factor in the short stature of the patients followed up at our growth outpatient clinic.

In addition to this, another inclusion criterion (that the children had to be more than two years old) also provides corroboration of the importance of birth weight in the prognosis for the height of these children, since there are reports in the literature that suggest that the majority of children that are small for the gestational age resume an adequate growth pattern, basically by the age of two years.^18-20^

Less than 15% of our patients with more than six months of follow-up, for whom it was possible to calculate the parental target, presented heights that were below the family's pattern. It is stressed that more than 50% of the patients' mothers also presented short stature, thus reinforcing the idea of the influence of the genetic factor. The average heights of our patients' mothers and fathers (153.6 cm and 165.9 cm, respectively) were less than the averages observed among a cohort born between 1966 and 1968 in Brazil (157.3 cm for women and 169.6 cm for men).^21^ However, the analysis of this influence is much more complex, because evaluation of these family members from a socioeconomic point of view would probably demonstrate that the correlation between the parents' and children's statures in cases of short stature has multiple causes.

When we analyzed the relationship between the growth rate (cm/year) and the bone age, we found that, as expected, most children and adolescents with adequate growth rates presented normal bone age, while those with inadequate growth rates presented abnormal bone age, thus drawing attention to the care that must be taken when coming across an abnormal result. In the light of a normal growth rate, such a result does not necessarily characterize a pathological slowness but, rather, it may highlight the growth potential that may be attained. The small number of patients with inadequate growth rates did not allow us to analyze the results from the bone age on these patients in a more detailed manner.

In analyzing growth as a continuous process, the importance of the growth rate in its evaluation is evident. However, as well as having definitions for standards, references and cutoff points, it is necessary to carefully measure the growth rate at different age bands. Terms like delayed or faulty growth are often utilized, although without specifying whether these refer to the short stature or a slow growth rate. Children may grow slowly for various reasons, such as growth hormone deficits, Turner's syndrome, hypothyroidism or celiac disease, and may also be above the cutoff percentile adopted.

A normal child tends to follow a pattern, i.e. one particular percentile, and deviations in growth away from this percentile are difficult to detect through observation of the growth curve. This is one of the reasons for calculating the growth rate.^22^

Two studies by Voss et al.^23,24^ that followed up children with short stature in the *Wessex Growth Study*, along with control cases of normal stature, observed that the growth rate did not distinguish between the children with short stature (varying from normality), the children with organ causes for their short stature, or the control cases. The authors of these studies preferred to recommend stature curves over growth rate curves for investigating inadequate growth, with the affirmation that the smaller the child is, the greater the prevalence of organ diseases will be. In the present study, the fact that one patient with a height of less than -4 z-scores presented short stature of familial/constitutional origin demonstrates the difficulty in characterizing the cause of short stature as a function exclusively of the observed z-score for height.

More recently, Cianfarani et al.,^25^ in a study among patients with growth hormone deficit and idiopathic short stature, noted that although the growth rate presented a sensitivity of over 80%, it showed insufficient specificity, since a growth rate of below P25 was observed in the majority of their patients with growth hormone deficiency and also in almost 60% of the children with idiopathic short stature. The growth rate therefore could not be utilized on its own for identifying the growth hormone deficit. They proposed that, in addition to the growth rate, the assaying of the IGF-I associated with a stimulus test is needed for diagnosing a growth hormone deficit.

The following question therefore arises: How should the growth rate be calculated and what cutoff point should be utilized?

One of the difficulties to be noted is that, if the growth rate is calculated on the basis of the difference between two measurements, it incorporates the imprecision of both readings. Measurement errors can result from inadequate techniques, variations between instruments and observers, diurnal variations, or incorrect annotation on the curves.^26^ It must also be emphasized that, while most doctors are familiar with growth curves for weight and height, few utilize growth rate curves.

The interval between the height measurements is another matter to be raised, and may be a factor for discussion. The growth rate curves were designed for intervals of 12 months between measurements, with the objective of adjusting for the seasonality of growth and keeping the measurement errors reasonably low. However, an interval of one year may represent a significant delay in the diagnosis and treatment of some diseases. The growth rate over any time interval can be assessed via the difference between the z-scores for height, with the use of an appropriate reference. Another way of calculating the growth rate as a function of the z-score is the so-called conditional gain in z-score for height, with the advantage that this allows regression to the average. This last parameter is more complicated to calculate and the difference in z-scores is clinically more practical and more utilized.^27^

Van den Broeck et al.^12^ analyzed the diagnostic validity of the growth rate (difference in z-score) over periods of one, two or three years, in order to differentiate growth disturbances. They found an overlap in growth rate over one year between normal children, those with growth hormone deficit and those with Turner's syndrome. The diagnostic validity increased when several years of follow-up were evaluated, and they proposed that, for pre-pubertal children, a difference in z-score ≤ -0.75 fora period of more than three years could be used as a criterion for future investigations.

In the present study, we proceeded with an analysis of the growth rate calculated in cm/year and in relation to the difference in z-score, among patients that continued with the follow-up for a minimum period of six months. We performed these two types of evaluation of the growth rate, in order to confirm whether or not the calculation in cm/year that was performed in daily practice would be capable of determining directions for investigation, so as to differentiate between etiologies and even to confirm whether this would be comparable to the difference in z-score.

In our survey of the literature, although we found studies that utilized the difference in z-score for evaluating the growth rate, thereby giving value to the concept of growth channeling, to our surprise these articles did not define what cutoff point should be adopted for this difference, for defining what growth rate was considered inadequate. Only the study by Van den Broeck et al.^12^ utilized a cutoff point and, while maintaining the due proportions and differences between the studies, we decided to utilize the same criterion (z-score ≤ -0.75) for comparison.

Among the 99 patients evaluated at the growth outpatient clinic, only two (one child and one adolescent) presented an abnormal difference in z-score, which made it impossible to proceed with the analysis according to this criterion. There is no doubt as to the importance of this manner of evaluating the growth rate, if only because the z-score index is increasingly indicated and utilized. For us, however, the cutoff point to be adopted remains an open question, particularly when dealing with longitudinal follow-up of patients with short stature.

Among the patients with growth rates of less than 4 cm/year, almost half of them (46.7%) did not complete one year of follow-up, which could be considered a bias in the evaluation of the growth rate among this group.

We can highlight some limitations observed in our study, among which the selection of the patients, in which the parents, the patients themselves or the doctor could determine this selection; and the difficulty in performing tests, especially for determining karyotypes and IGF (insulin growth factor) values that could guide the diagnostic definition of short stature.

In addition to this, just as in some of the studies described above, the growth rate in itself was not capable of guiding the diagnostic investigation, since we observed patients with adequate growth rates but abnormal X-ray for bone age assessment, and with one or even two non-responsive growth hormone stimulus tests. On the other hand, some patients who were considered to present short stature of familial/constitutional origin presented inadequate growth rates.

## CONCLUSIONS

It is concluded that growth rate assessment must form part of the investigation and follow-up of short stature. However, its utilization and validity should form part of the analysis of a set of factors: anamnesis, physical examination, X-ray for bone age assessment and other complementary tests, with the aim of building up an overall view of each patient with short stature.
